# Promotion and tenure policies for team science at colleges/schools of medicine

**DOI:** 10.1017/cts.2019.401

**Published:** 2019-10-08

**Authors:** Susan M. McHale, Damayanthi (Dayan) Ranwala, Deborah DiazGranados, Dee Bagshaw, Erich Schienke, Arthur E. Blank

**Affiliations:** 1 Human Development and Demography, Social Science Research Institute, Penn State Clinical and Translational Science Institute, The Pennsylvania State University, University Park, PA, USA; 2 Department of Psychiatry and Behavioral Sciences, Medical University of South Carolina and Pilot Project Program and Team Science Program, South Carolina Clinical and Translational Research Institute, Charleston, SC, USA; 3 School of Medicine and Evaluation and Team Science at the Wright Center for Clinical and Translational Research, Virginia Commonwealth University, Richmond, VA, USA; 4 Penn State Clinical and Translational Science Institute, The Pennsylvania State University, University Park, PA, USA; 5 Department of Energy and Mineral Engineering, Penn State Clinical and Translational Science Institute, The Pennsylvania State University, University Park, PA, USA; 6 Departments of Family and Social Medicine and Epidemiology and Population Health, Evaluation, Harold and Muriel Block Center for the Evaluation of Translation Research, Albert Einstein College of Medicine, New York, NY, USA

**Keywords:** Team science, interdisciplinary, collaboration, promotion and tenure, academic career

## Abstract

**Introduction:**

Advancing understanding of human health promotion and disease prevention and treatment often requires teamwork. To evaluate academic medical institutions’ support for team science in the context of researchers’ career development, we measured the value placed on team science and specificity of guidance provided for documenting team science contributions in the promotion and tenure (P&T) documents of Colleges/Schools of Medicine (CoMs) in the National Center for Advancing Translational Sciences’ Clinical and Translational Science Award (CTSA) program.

**Method:**

We reviewed complete P&T documents from 57 of 63 CTSA CoMs to identify career paths defined by three dimensions: academic rank (associate versus full professor), tenure eligibility (tenure track versus not), and role (research, clinical, education, and administrative), and we rated team science value and documentation guidance for each path. Multilevel models were estimated to compare team science value and documentation guidance as a function of the three career path dimensions while accounting for the clustered data (*N* = 357 career paths within 57 CoMs).

**Results:**

Team science value was greater for associate than full professors, non-tenure-eligible versus tenure-eligible positions, and roles prioritizing clinical, education, and administrative responsibilities versus those prioritizing research. Guidance for documenting team science achievements was more explicit for roles that prioritized research.

**Discussion:**

Although P&T policies at most CTSA institutions express value for team science, inconsistent within-institutional patterns of recognition and reward across career paths may have implications for researchers’ involvement in team science. We discuss the implications of our findings for research and for P&T policies that promote team science.

## Introduction

Increasing specialization of scientific knowledge, in combination with increasing complexity in health and health care problems, underlies the growth of and necessity for team science in academic medical institutions [1–3]. Indeed, a growing body of research documents that team science – collaborative, often interdisciplinary research aimed at addressing complex problems [4] – has been linked to impact, such as in paper citations and patents and accelerating innovations, including in the domains of biomedical and health sciences [5–7]. Team science and the competencies it requires, however, have not typically been the focus of graduate and medical education. And, although team science concepts may initially seem like common sense, their practice is often challenging [1]. One such challenge lies in the alignment of institutional incentives for team-based collaborative research [8–11].

Within industrial organizational psychology, theories of motivation that focus on expectancies assert, and empirical studies document, that individuals are motivated to align their work activities with the reward system of their work organization [12]. Although promotion and tenure (P&T) policies are at the foundation of the reward system in the academy [9–11, 13], they are reported to be one of the most serious impediments to team science [10, 13, 14]. In response to this concern, a growing number of reports outline recommendations on best practices for institutional support of team science in the P&T process [8–11, 13, 14].

Empirical research on P&T policies pertaining to team science, however, is limited. One early study analyzed data from the 2005 Faculty Personnel Policies Survey conducted by the Association of American Medical Colleges, which focused on 125 Colleges/Schools of Medicines (hereafter referred to as CoMs) accredited by the Liaison Committee of Medical Education [15]. Findings revealed that, between 2002 and 2005, 15 (12%) of these institutions modified their P&T policies to highlight the significance of interdisciplinary team science and 24 more (19%) were currently deliberating about including such language. This analysis, however, did not provide information on the total number of CoMs that included language about the value of team science in their policy documents at that time. More recently, a qualitative analysis of complete P&T policy documents from 18 research-intensive Colleges/Schools of Nursing revealed that only 8 (44%) included any references to team science, and those references were, in the authors’ words, “largely negligible” (p. 1) [16].

The most widely cited study examined the P&T policies of CoMs that were supported by the Clinical and Translational Science Award (CTSA) program of the National Center for Advancing Translational Science (NCATS) [17, 18]. The CTSA mission emphasizes the significance of team science toward increasing the impact of research on human health [3], suggesting that these institutions should be at the forefront in efforts to promote team science. The investigators surveyed administrators (e.g., assistant/associate deans; deputy/vice provosts) at 60 CTSA institutions in 2012 and requested excerpts from P&T policy documents that included reference to collaborative, inter/multidisciplinary, and/or team science. Of the 60 institutions surveyed, 43 (73%) responded and, of those, 10 reported no references to team science. Qualitative analyses of the 33 excerpts received revealed that only 18 (54% of those that provided excerpts; 42% of those responding) included explicit recognition of team science and only 2 included team science in its definition of scholarly excellence. Based on these results, the investigators argued for the importance of outreach to academic institutions to clarify and enhance their P&T policies in support of team science [13, 17, 18]. The study’s findings have been referenced in seminal reports aimed at promoting support for team science including by the Canadian Academy of Health Sciences [19], the United Kingdom’s Academy of Medical Sciences [20], and in the National Research Council’s handbook, *Enhancing the Effectiveness of Team Science* [10]. Based on these data and an extensive literature search for reports on team science and P&T, Klein and Falk-Krzesinski developed a framework for P&T policies aimed at promoting “consistency, alignment, and comprehensiveness in creating a culture of reward” (p. 1056) for team science [9].

Discussions of approaches to promoting team science have also directed attention to policies and practices for documenting candidates’ team science contributions in the context of P&T decisions [8–10]. The analyses of P&T policy excerpts described above revealed that most (*N* = 27; 82% of institutions that provided excerpts) included criteria for team science involvement but less than half (*N* = 16; 48% of participating institutions) provided guidelines for candidates in documenting their team science contributions [17, 18]. The lack of guidelines for documenting team science contributions contrasts sharply with established criteria for determining independent science contributions, which include, for example, numbers of first and senior author publications and principal investigator (PI) roles on grant awards. Not only are team science-focused researchers handicapped when they lack well-defined criteria for documenting their contributions but also the lack of explicit achievement metrics can result in greater subjectivity and even bias in P&T reviews and decision-making [8, 9, 14]. Providing guidance and associated metrics for team science achievement in the context of P&T policies can clarify how faculty who are immersed in team science achieve success; such guidance may also convey the centrality of team science for advancing knowledge and practice and serve to communicate the institution’s value for team science [8, 9]. Recognition of the importance of clear and consistent guidelines has been the basis for recommendations for documenting team science achievements [9, 14] and is exemplified in descriptions of the P&T policies of specific institutions [10, 21] and in the guidelines for conveying individuals’ contributions to team science by a range of professional organizations [22] and publication outlets [23, 24]. Systematic research aimed at documenting whether and how institutions are responding to such calls for clear and consistent documentation guidance, however, is limited to one prior study [17, 18].

The current study was designed to address the limitations of the empirical literature on P&T policies for team science. The few prior studies were based on administrators’ reports of whether or not P&T policies at their institution included team science language [15, 17, 18], relied on excerpts from policy documents that were chosen by administrators [17, 18], and/or used qualitative analyses to provide descriptive information about the recognition or value placed on team science and practices for evaluating a faculty member’s team science contributions [16–18]. The results primarily document the number of institutions with (and without) particular policy language, and we thus know little about the larger institutional contexts in which these policies are embedded. Academic institutions are complex organizations and include faculty members with a variety of roles and responsibilities [10]. Indeed, analyses of 25 years of change in US CoMs revealed an increasing number of faculty career paths within CoMs as one of the major developments in these institutions: between 2002 and 2005 alone, 27 (22%) of the 125 CoMs surveyed added new career paths in an effort to account for the range of faculty responsibilities [15]. As such, in any given institution, P&T policies may not be monolithic, but rather may vary depending upon the terms of faculty members’ appointments. Importantly, although new career paths may be developed in an effort to promote team science, such within-institution variation may be another kind of obstacle to advancing team science if status and rewards within the institution differ for team versus independent scientists. Further, to the extent that the value placed on team science differs across career paths within a CoM, the institution may be providing a mixed message about its significance. This is especially so if team science is less highly valued in higher status positions such as tenured full professor.

Accordingly, to shed new light on the role of P&T policies in promoting team science, we expanded on prior work to address four goals: (1) Identify distinct career paths as defined by *rank* (associate versus full professor), *tenure eligibility* (not-tenure-eligible versus tenure-eligible track), and *role* (research prioritized, clinical or education responsibilities prioritized, administrative/other responsibilities prioritized) based on reviews of complete P&T documents. We focused on career paths within the CoMs at CTSA institutions given the CTSA mission to promote team science [3], though these issues are relevant to CoMs more generally as well as to disciplines beyond the biomedical and health sciences; (2) Evaluate both the value placed on team science and the specificity of guidelines for documenting team science contributions for each career path; (3) Using a quantitative approach, test whether the ratings of team science value and the specificity of guidelines for documenting team science achievements differed as a function of career path dimensions (i.e., rank, tenure eligibility, and/or role); (4) Explore whether tested institution-level factors including CoMs’ public versus private status, *US News* rating, and date of P&T policy accounted for between-institution differences in team science value and/or documentation guidance.

## Materials and Methods

### Sample

Four study authors (SM, AB, DDG, DR) are members of the CTSA Methods and Processes Domain Task Force’s Institutional Readiness for Team Science Working Group (WG). We began by inviting WG members, who represented CTSA institutions, to provide the current P&T documents for the 2016–2017 academic year from their institution’s CoM. For CoMs without WG representation, we sent a letter to the CTSA PI requesting a copy of the CoM’s P&T policy documents. We received complete P&T policy documents from 49 of the 63 institutions that were invited to participate (79%). We expanded the sample by searching online for policy documents from non-responsive CoMs and found policy documents from eight additional institutions. Documents from the remaining five institutions were not publicly available. Thus, our final sample included documents from 57 CTSA institutions (92% of institutions invited). Most policies were extensive. Some were embedded in the CoM’s faculty handbook, some included different documents for faculty with different roles (e.g., research-focused faculty; clinical educator faculty), and some provided links to additional details about P&T policies and practices. Most policies (60%) were dated after 2014, but 22% were dated between 2010 and 2014, and 13% were dated prior to 2010. We could find no date for an additional 5%.

### Procedures

In a preliminary step, two co-authors (SM and DB) reviewed documents from a randomly chosen 12 CoMs to identify criteria for tenure and promotion to associate and full professor ranks. Based on reviews of all P&T materials provided by these institutions and on prior literature [17, 18], we chose two dimensions for analysis, the *value for team science* and level of *guidance for documenting team science achievements*, and we created rating scales to assess these dimensions (Table 1). Next, three co-authors (SM, DB, and ES) reviewed each CoM’s documents to identify its *career paths*, as defined by three dimensions, *rank* (associate versus full professor), *tenure eligibility* (tenure track versus non-tenure track), and *role* (research, clinical, education, administration, and other). Career paths that involved no research responsibilities were excluded from further analysis (given that these faculties would not be evaluated for their science achievements); although teamwork may be valued in the scholarship of education and practice, our focus here was on team science in career paths that included research responsibilities.

**Table 1.** Definitions and verbatim examples of codes for team science value and specificity of guidance for documenting team science achievement

Variable	Values	Definitions and examples from P&T documents
**Team science value**	1 = Independent science only	Criteria are specific to independent science, including the significance of first/senior author, PI; no mention or statement of support for team science. For example: “An ongoing, peer-reviewed publication record with first- or senior-author publications” “Independent funding and reasonable expectation of continued independent funding”
	2 = Independent science but may be involved in team science	Criteria are focused on independent science but provide some support for team science including that the dossier can include middle authorship or co-investigator status. For example: “Typically 1–2 publications on average per year as first or senior author … although consideration is also given to … faculty whose work is primarily part of team research” “Co-investigator status may be a recognizable measure of responsibility and activity”
	3 = Independent and team science valued equally	Criteria balance value for independent and team science. For example: “Continuing publications in peer-reviewed journals of high quality providing evidence for substantial contributions to a field, including an important collaborative role in these efforts” “Sustained independent funding or plays a documented role in obtaining and maintaining funding for collaborative efforts”
	4 = Team science valued most strongly	Criteria are focused on collaborative-/team-based research; policy highlights expectations for team science. For example: “The primary focus … is to facilitate and support the overall research mission of [Institution name], rather than to develop independent programs…. faculty typically conduct research in collaboration with other investigators or groups of investigators” “Authorship that is not “first” or “senior” may be highly regarded in the evaluation of a candidate.”
**Specificity of guidance for documenting achievements**	1 = No guidance provided	No mention of documenting team science contributions
	2 = Documentation required but no guidance	Documentation of team science contributions is required but no specific guidelines or metrics are provided. For example: “Team-based investigation is also recognized …. However, for those individuals engaged primarily or exclusively in collaborative research, it is imperative that the individual’s contribution to collaborative efforts be clearly outlined in the dossier, with documentation of innovation and leadership in their own area.” “A narrative personal statement must be submitted … this narrative is limited to two pages in length and should explain the contribution to scholarship or other accomplishments without recapitulating the curriculum vitae.”
	3 = Details provided on how to document team science achievements	Documentation is required; specific guidelines/measures in documenting the team science are provided. For example: “Team candidates should annotate each team publication and team grant on their CV to indicate the precise role and the nature and extent of the contribution they made to that publication or research” “At least two of the four collaborators mentors/colleagues selected to write on behalf of the candidate should be identified as a Team Colleague, and one of these should be the team’s leader. Such referees will be explicitly asked to address the question of the candidate’s contributions to team science”

CV, curriculum vitae; PI, principal investigator ; P&T, promotion and tenure.

The policy documents sometimes provided a general statement of the CoM’s value for team science, but we focused on the expectations for accomplishments that were provided for specific career paths: Our team reviewed each CoM’s policies pertaining to each of its identified career paths and rated the *value for team* science using a four-point ordinal scale, with higher scores signifying greater value and *guidance for documenting team science achievements* using a three-point scale, with higher scores signifying more detailed and explicit guidance for how to document team science achievements (see Table 1 for example excerpts). Beginning with *Team Science Value*, a score of 1 was coded if the policy focused on independent achievements such as senior author and PI roles as criteria for tenure and/or promotion and failed to include any language that conveyed value candidates’ involvement in collaborative research/teams. A score of 4 was coded if the policy specified that involvement in team science was the criterion for achievement and no reference was made to independent achievement as an expectation for tenure and/or promotion. Between these two ends of the coding continuum, a score of 2 was coded if criteria highlighted independent achievement but noted that team science achievements might also be considered, whereas a score of 3 was coded if criteria placed equal emphasis on independent (e.g., first author, PI) and team science (e.g., middle author, co-investigator) achievements. In terms of *Specificity of Guidance* for documenting team science achievements, also shown in Table 1, a score of 1 was coded if there was no mention of the documentation process; a score of 2 was coded if there was a stated expectation that the candidate’s contributions to research teams should be documented; and a score of 3 was coded if the policy included the expectation for documenting team science contributions as well as specific kinds of documentation that should be included in the candidate’s P&T materials.

At least three members of the coding team independently rated team science value and team science documentation guidance for each career path, for each CoM. In bi-weekly conference calls, we used a consensus agreement approach [25] to determine final scores for each of these two dependent variables for each career path in each CoM.

### Analyses

We first calculated descriptive statistics for the dependent variables, team science value, and documentation guidance. Next, to take into account the clustered design (career paths within CoMs), we tested differences in team science value and in documentation guidance (one model for each dependent variable) as a function of rank (1 = associate), track (1 = tenure eligible), and role (dummy variables for clinician/educator and other; research-focused as the reference group), by estimating multilevel models using the nlme package in R 3.5.0, https://CRAN.R-project.org/package=nlme. Level 1 of these models captured factors that varied within institutions, namely, rank, tenure eligibility, and role. At Level 2, we entered factors that captured between-institution differences, namely: year of policy (1 = before 2009; 2 = 2010–2013; 3 = 2014 and later), public versus private institution status (1 = public), and the CoM’s *US News* ranking as additional covariates. Given that the source of the policy documents varied, we also included this factor (1 = policy obtained online) as a between-institution control variable at Level 2. We began by testing model fit to determine whether random effects should be included in the model for each dependent variable. We also tested two- and three-way interaction terms involving rank, tenure eligibility, dropping those interaction terms that proved non-significant [26]. Using the team science-dependent variable as the example, the model equation was as follows:

Level 1: 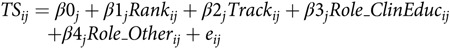

Level 2: 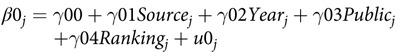

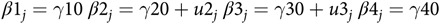

where *ij* is the *i*th career path at CoM_*j*_.

Combined equation: 
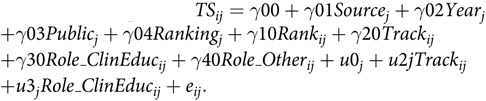


Random effects: 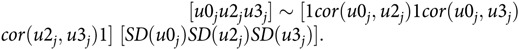


## Results

### Descriptive Analyses

We identified *N* = 357 career paths across the 57 CoMs for coding. CoMs varied widely in the number of career tracks that included at least some research responsibilities: one institution included only one career path with research responsibilities, one included 14 such career paths, and the mean (*M*) across CoMs was 6.26 (*SD* = 2.77) paths, suggesting the possibility of substantial within-institution variation in P&T policies. Indeed, intraclass correlations (ICCs) were suggestive of within-institution variation, especially in team science value, ICC = 0.36, and less so in documentation guidance, ICC = 0.85. A slight minority of career paths were tenure eligible (45%), and most paths that included any research responsibilities prioritized research (65% of paths) followed by prioritization of clinical and/or educational (32%) and administration/other (3%) roles. Proportions of paths at the associate and professor rank were approximately equal. Importantly, however, in addition to research, some education and service/practice activities were expected for almost all paths. Descriptive analyses also revealed that the P&T policies of most (95%) institutions expressed some support for team science for one or more paths (i.e., team science value greater than 1 on our four-point scale; *M* = 2.41, *SD* = 0.65). Guidance for documenting team science achievement was slightly below the scale midpoint, *M* = 1.83 (*SD* = 0.77). Approximately 54% of the CoMs’ policies (31 CoMs) included no documentation guidance for one or more of their career paths. Finally, bivariate correlations indicated that value for team science was positively and moderately associated with clarity of documentation guidance, *r* = 0.33, *p* < 0.001.

### Differences in Value and Guidance for Documenting Team Science Achievements

Results are shown in Table 2. Model testing revealed that the best fitting model included random effects of track (tenure eligibility) and role (research versus clinician/educator) for both the analyses of team science value and documentation guidance, and thus these effects were included in the final models. Tests of two- and three-way interactions revealed no significant effects, so these were excluded from the models.

**Table 2.** Results of multilevel models predicting team science value and specificity of guidance for team science documentation

	Team science value		Team science documentation guidance	
**Fixed effects**, ***γ*** **(*****SE*****)**				
Intercept	1.32	(0.34)***	0.95	(0.41)*
Source (1 = policy online)	0.64	(0.23)**	0.03	(0.29)
Year (1 = before 2009; 3 = after 2014)	0.09	(0.10)	–0.09	(0.12)
Rank (1 = associate)	–0.10	(0.05)*	–0.01	(0.02)
Tenure track (1 = tenure eligible)	0.62	(0.13)***	0.03	(0.07)
Public	–0.08	(0.18)	0.14	(0.22)
Role clinical educator (1 = research focus)	0.28	(0.10)**	–0.13	(0.03)***
Role other (1 = research focus)	0.65	(0.29)*	–0.01	(0.15)
College ranking	0.01	(0.01)	–0.01	(0.01)
**Random effects with** ***CI*****s**				
*SD*(Intercept)	0.78	[0.61, 0.99]	0.81	[0.66, 0.99]
*SD*(Tenure track)	0.88	[0.69, 1.12]	0.50	[0.40, 0.63]
*SD*(Role_ClinEduc)	0.47	[0.31, 0.71]	0.48	[0.21, 1.10]
*Cor*(Intercept, Tenure track)	–0.66	[–0.82, –0.42]	–0.31	[–0.55,–02]
*Cor*(Intercept, Role_ClinEduc)	0.53	[–0.08, 0.85]	0.35	[–0.42, 0.83]
*Cor*(Path, Role_ClinEduc)	–0.40	[–0.75, 0.12]	–0.99	[–1.00, 0.81]
**Fit statistics**				
AIC	746.14		316.36	
BIC	807.77		378.00	
–2 Log likelihood	714.14		284.36	

AIC, Akaike information criterion; BIC, Baysian information criterion; *γ*, *coefficient*; *CI*, confidence interval; *Cor*, correlations for random effects; *SD*, standard deviation for random effect; *SE*, standard error.

*p < 0.05, **p < 0.01, ***p<0.001.

Beginning with *team science value*, a significant effect emerged for rank, indicating that team science was valued more strongly for associate professors than full professors, and a significant effect also emerged for tenure eligibility, indicating that team science was valued more highly for non-tenure-eligible than tenure-eligible faculty. Table 3 provides example excerpts on the value of team science for tenure eligible and non-tenure-eligible roles in the same CoM. Comparisons as a function of role also revealed significant effects: team science was valued more strongly for roles that prioritized clinical and educational responsibilities and roles that prioritized administrative and other responsibilities as compared with roles that prioritized research (the reference group). Among the institution-level factors, the only significant effect on team science value was source of policy documents: CoMs that provided us with their policy documents scored higher on value for team science than those whose policies we located online.

**Table 3.** Example policy excerpts describing expectations for independent versus team science for tenure-eligible versus non-tenure-eligible tracks within the same institution

Tenure-eligible	Non-tenure eligible
*“… demonstrate a career commitment to scholarly pursuit and have documentation of their endeavors by way of significant publication, grant support, peer recognition for outstanding research national and international recognition.”*	*“an important supportive role in the genesis, conduct and reporting of research findings; considered an essential member of the team carrying out the research in a supportive or fundamental role and may be a PI, Co-PI… Co-Investigator or Key Personnel on funded grants”*
*“… expected to develop an extramurally funded program of research that is internationally recognized…. Such research should be original, creative, transformative, and it should substantively advance the discipline of the faculty member … prepared to seek independent, peer-reviewed research support.”*	*“Research funding as co-investigator from federal, foundation, or industry resources … Authorship on multi-authored journals articles and/or documentation of a major, substantial contribution by the candidate to a collaborative, multidisciplinary project and publications.”*
*“Candidates must have a national reputation for outstanding independent work in their area of scholarship … [candidate] will have a history of having been awarded several independent research grants…”*	*“…faculty provide critical expertise to a program or group of research teams as a co-investigator with contributions that do not necessarily require or result in independent grant funding, but some faculty on this track may serve as principal investigator on related research …”*
*“Leader … original papers that must clearly highlight the individuals’ role in advancing the field; investigator driven [grants], most as PI or one of multiple PIs”*	*“Collaborator and sometimes leader… original papers as either a project leader or collaborator … collaborative and sometimes investigator-driven”*

Co-PI, co-principal investigator; PI, principal investigator.

Turning to *guidance for documentation of team science achievements*, one significant effect emerged for role: More specific guidance on documentation was provided for roles that prioritized research than for roles that prioritized clinical and education responsibilities. Institution-level covariates were non-significant in this model.

## Discussion

Our study contributes to a small empirical literature aimed at evaluating academic institutions’ support for team science in P&T policies. Building on prior research [15–17], we reviewed complete P&T policy documents from 57 CoMs in the CTSA Consortium – given NCATS’s goal of advancing team science [3] – toward illuminating elements of institutional culture surrounding team science. Academic medical institutions are complex organizations that incorporate an increasing number of career paths [15]. This novel insight was the basis for this study’s goal of assessing potential *within-institution variability* in the value placed on team science and the guidance provided for documenting team science achievements in a CoM’s P&T policy documents as a function of researchers’ rank, tenure eligibility, and institutional role.

Results revealed that, in general, P&T policies expressed *value for team science*: As noted, the cross-institution mean fell above the midpoint on our four-point rating scale. This overall average, however, masked significant variability as a function of candidates’ academic rank, tenure eligibility, and role: Our analyses of team science value scores for the 357 career paths that we identified revealed that team science was valued more highly for associate as compared to full professors, non-tenure as compared to tenure-eligible roles, and paths that involved primarily clinical and education as compared to research responsibilities. We found no effects for year of policy, *US News* ranking, or public versus private institution status, but CoMs that provided P&T documents scored higher on team science value than those we obtained from our online search.

Over a decade ago, Bunton and Mallon reported a trend among CoMs to develop new career paths in an effort to address a growing range of faculty roles and responsibilities [15]. Consistent with this trend, our results revealed that CTSA CoMs averaged over 6 career paths and that, among the policy documents we examined, as many as 14 paths within a single institution included a research role. Creating new career paths with different criteria for advancement is one way of accommodating and acknowledging the distinctive accomplishments of team scientists. Indeed, five of the institutions we studied included career paths specifically designated for researchers engaged in team science. Career paths that emphasized team science, however, were less like to be tenure-eligible.

The variability of P&T policies across career paths in the CTSA’s CoMs may thus create a quandary for potential team scientists. Our results suggest that, if becoming a tenured full professor in a research-focused role is a career goal, working as a team scientist may undermine researchers’ ability to achieve this goal. Variation in the value for team science across career paths may also have implications for the institutional culture to the extent that a lesser value is placed on team science for those in higher status career positions, namely, tenured full professors with a primary responsibility of advancing science. Published recommendations about P&T policies have stressed that consistent messages about the value of team science are essential to developing a culture that promotes and supports team science [9, 10]. Our findings revealed an added nuance – *establishing within-institution consistency across career paths –* is a key step toward this goal. Even if team science by researchers in some career paths is highly valued by an institution, to the extent that researchers who are primarily involved in team science are lower status in terms of role, rank, and/or tenure eligibility, *institutions may be conveying a mixed message about the value they place on team science*.

As others have argued, and consistent with expectancy and motivation theories [12], incentives within the P&T system must be aligned with the expressed value of team science if the goal of promoting team science is to be achieved [9, 10]. We argue that success is unlikely if team scientists are seen or treated as second class scholars in the P&T process. Thus, a key next step is to assess faculty perceptions of the *status and institutional rewards* specific to each of their institutions’ many career paths. If it is indeed the case that paths prioritizing independent science are perceived to be higher in status and rewards – as our findings suggest they might be – there may be long-term implications for the quality of team science because the most talented researchers may choose higher status/reward career paths. Thus, we recommend that CoMs review their P&T policies to determine whether team science achievements are differentially valued across career paths defined by rank, role, and tenure eligibility, and if they are, work to align criteria across career paths to provide a *consistent, cross-institution message* on the value of team science.

Our findings on differences in the value placed on team science as a function of rank are relevant to the concern [13, 27] that P&T policies promote “Individual reputation first, collaboration later” (p. 185) [10]. As we have noted, however, in contrast to this maxim, our results suggested that P&T policies may be sending another mixed message about career advancement. We found that, although a record that includes team science is acceptable early in faculty members’ careers as they advance in rank from assistant to associate professor, advancement to full professor more often requires independent science credentials. This pattern – support for team science conducted by junior faculty in the face of requirements for independent science for promotion to the full professor level – may undermine the institution’s stated value for team science. First, it may have the unintended consequence of limiting promotion opportunities for researchers who begin their careers as team scientists because they lack the independent science credentials necessary for promotion. Indeed, one published recommendation for promoting career development in clinical and translational science trainees is that interdisciplinary scholars be explicitly advised to balance publications that reflect interdisciplinary team science with those that evidence their independent science [14]. This pattern may also serve to diminish an institution’s stated value for team science – because of perceptions that team science is valued primarily for researchers who are lower in rank. Such concerns underscore our recommendation that institutions examine their P&T policies to align criteria and provide a consistent, cross-institution message on the value of team science. From an organizational perspective and consistent with motivation theory, academic institutions can create environments that elicit desired behaviors by aligning those behaviors with rewards, such as high-status roles [12].

Importantly, none of the P&T documents we examined included policies for reviewing established (tenured full professor) investigators – those who, having established themselves as independent scientists, may be most free to pursue team-based research. To our knowledge, there are no empirical studies of policies for reviewing established investigators; this would be another step toward understanding institutional supports for team science. We recommend that P&T policies include clear criteria for post-tenure review so that researchers within an institution are informed about their institution’s value for team science across their research careers.

Turning to *guidance for documenting team science achievements*, our findings also revealed inconsistent messages. Here, however, we found only one statistically significant effect: More explicit guidance was provided for roles that emphasized research compared to roles that emphasized clinical or educational responsibilities. On one hand, this finding suggests that CoMs have heeded prior calls for clarity and detail in how to best make the case for team science achievements [8, 9]. There are other less positive implications, however. When specific documentation of team science contributions is required only for research-focused candidates, an extra burden is placed on these team scientists in developing their P&T dossiers. An unintended consequence of this inconsistency may be a message that the most straightforward path for a researcher is to pursue a traditional, independent science-focused career. Inconsistency in policies around documentation may also suggest that, whereas the team science activities of candidates with primarily clinical or educational responsibilities are normal and expected, those of candidates with primarily research responsibilities are not and thus require special explanation. As others have argued, institutions should provide consistent metrics and clarity about the process of documenting team science both to inform faculty development and to legitimize team science [8, 9]. Our findings suggest that this recommendation be qualified to add that institutions examine their P&T policies to align their guidance for documenting criteria for team science achievements to provide *consistent intra-institution expectations*.

In the face of our study’s contributions, its limitations provide directions for future research. By reviewing complete policy documents, we were able to move beyond general statements about CoMs’ value for team science to code policies relevant to particular career paths within each institution, shedding new light on the CTSA CoM’s support for team science. But, policy may differ from practice. That is, formal policy may mask informal practices that surface when a candidate’s promotion package is placed before a P&T review committee. For example, team science may be evaluated more positively in informal processes than is conveyed in the P&T documents we reviewed. Although highly sensitive, analyses of reports from review committee members and administrators on how they consider team science contributions in their evaluations will provide additional insights into CoMs’ support for this enterprise.

Further, although our findings on inconsistency across career paths in the value placed on team science suggest that researchers may be receiving mixed messages from their institutions, as noted, we did not measure such subjective experiences. Researchers on a team science track may perceive their institution as placing a strong value on team science because such a career path has been established even though they are not, for example, tenure eligible. Research focused on researchers’ perceptions of their institutions’ value for team science is essential to understand whether and how within-institution variation in P&T policies for team science has implications for the institutional climate and for the career decisions and plans of individual researchers to engage in team science.

Importantly, our findings are limited to CTSA institutions, and given CTSA’s support and promotion of team science, team science value and documentation guidance may be stronger at these institutions than at CoMs in general. On the other hand, we tested both US News rankings and public/private status as potential covariates of team science value and documentation guidance and neither proved a significant predictor, suggesting that our findings have some generalizability. Future research, however, should be extended to a broader range of CoMs as well as to focus on disciplines beyond biomedical and health researchers if we are to understand the challenges facing broadly interdisciplinary team science.

Future research should also be directed at examining whether the proliferation of career tracks that appears to underlie within-institution variability in P&T policies has implications for team science excellence and impact. As others have argued, it would be a mistake to promote team science for its own sake because, just like independent science, the quality of the research stemming from team science is likely to be highly variable [28]. Indeed, investigators engaged in the science of team science are providing important insights into the conditions under which and processes through which team functioning is optimized [8, 9, 28–30]. Research is sorely needed to test whether and how P&T policies promote excellence and real-world health impact in the contributions emanating from clinical and translational team science.

Translational science is a team sport [3], and given their mission, the CTSA CoMs can serve in a leadership role to address the factors that support and promote team science excellence [2]. The CTSAs, and academic institutions more broadly, may sit at a crossroad. Researchers engaged in the science of team science have developed a body of evidence ranging from studies of effective team functioning to analyses of institutional policies, to which our study contributes. Advancing the science of team science would be furthered by explicit calls by NCATS and other of the National Institutes of Health. Such funding would allow team science researchers to put existing knowledge to use in research that systematically evaluates the conditions under which team science achieves its potential to improve the health of patients and populations.

## Previous Presentations

Portions of this work were presented at the Association for Clinical and Translational Science meeting, April 2017 and April 2019, Washington, DC.
